# Perceptions of Participants in a Group, Community, Exercise Programme for People with Multiple Sclerosis

**DOI:** 10.1155/2015/123494

**Published:** 2015-09-27

**Authors:** Rosemary Clarke, Susan Coote

**Affiliations:** Department of Clinical Therapies, University of Limerick, Limerick, Ireland

## Abstract

*Purpose*. The purpose of this study was to explore the perceptions of people with multiple sclerosis of a community based, group exercise programme. *Method*. A pragmatic programme evaluation approach using qualitative research design was adopted. Focus groups were used to gather data from 14 participants who had taken part in a RCT of community based exercise interventions for PwMS who used at most a stick to walk outdoors. Data were transcribed verbatim and thematic analysis was used to first identify categories and then to group them into themes. *Results*. Three themes emerged, psychological benefits, physical benefits, and knowledge gained. The psychological benefits included the role of the group as a social and motivational factor, empowerment, confidence, hope, sense of achievement, and pride. Physical benefits were improved energy and reduced fatigue and improved ability and participation. Knowledge gained caused a shift from thoughts that exercise might do harm, to sufficient knowledge that would give participants confidence to exercise themselves. The role of the group was a key element in the positive outcomes. *Conclusions*. The qualitative analysis supports the findings of the main trial confirming positive effects of community exercise interventions by reducing the impact of MS and fatigue and improving participation.

## 1. Introduction

Multiple sclerosis (MS) is a chronic debilitating disease of the central nervous system (CNS). It is characterised by the two simultaneous processes, inflammation leading to demyelination and degeneration of neuronal axons, resulting in the disruption of axon potentials in the brain and spinal cord [1]. Depending on the area of the CNS affected MS can cause a multitude of motor, sensory, visual, psychological, sexual, and bladder and bowel symptoms.

While there have been significant advancements in the range and efficacy of pharmacological interventions to reduce the number and severity of relapses, there remains no cure for MS. It is therefore essential that we develop and evaluate interventions that reduce symptoms and improve quality of life for people living with MS. Exercise is one modality for which there has been a significant paradigm shift over the last 2 decades. Previously it was thought that exercise may exacerbate symptoms; however, we now have an ever increasing body of evidence to suggest that it is not only safe but also effective at many levels [2–7]. Meta-analyses on the effect of exercise found that cumulatively there is a positive effect on QOL [8, 9] and walking mobility [10]. Additionally, there is increasing evidence for the effectiveness of exercise on fatigue [11, 12] and depression [13].

Qualitative studies can be a useful way of exploring the benefits of exercise programmes from the perspective of participants. Several previous qualitative evaluations of exercise for people with MS were found. Dodd et al. [14] used semistructured interviews of people who completed their 10-week progressive resistance exercise training, while Learmonth et al. [15] and Aubrey and Demain [16] used focus groups evaluating community exercise programmes. The common themes emerging from these papers' data were that physical, psychological, and social benefits were derived from the programme and that the group setting had many positive aspects. Participants also reported reductions in fatigue and feeling stronger at the end of the programme. Smith et al. [17] explored the influence of exercise on fatigue perceptions in people with MS who took part in an individual aerobic and strengthening programme for 8 weeks. Their participants perceived both positive and negative influences of the intervention on fatigue. These qualitative studies added to the quantitative findings and provided a richer perspective about the outcome of the intervention.

The purpose of this study was to explore the perceptions of participants of 10 weeks of group exercise in the community which was part of a multicentre, randomised, and controlled trial known as Getting the Balance Right [18]. The addition of a qualitative evaluation to quantitative measures in a RCT is advocated by the Medical Research Council in their guidance on evaluating complex interventions [19]. In this case, the aim was that the qualitative data would assist in understanding factors affecting participation, explore other effects not captured by the quantitative data, and would aid implementation into everyday practice through an understanding of the intervention from the perspective of users.

## 2. Study Design

A pragmatic programme evaluation approach using qualitative research design was adopted. Focus groups were used as a tool to gather the opinions of a selection of participants from the main study. Focus groups are particularly useful as they allow participants to clarify and distil ideas during the process as well as enabling them to voice opinions and raise aspects not previously considered by the researcher [20, 21].

Purposeful sampling was employed to identify participants in the RCT who attended programmes in Limerick during the time frame of the study. Information leaflets were distributed either in person at the postintervention quantitative assessment or by post. Those interested consented to sharing their contact details with the investigator who then telephoned them to schedule an appointment.

The participants in this qualitative study all used at most a stick to walk outdoors (Guy's Neurological Disability Scale [22] mobility section score of 0, 1, or 2) and were block randomised to exercise in groups in the community led by physiotherapists or fitness instructors. The protocol for the RCT has been described in detail previously [18]. Both physiotherapy and fitness instructor interventions consisted of similar combined strength and aerobic exercise, in the community, once weekly for 10 weeks.

## 3. Participants

A convenience sample of 14 individuals agreed to participate in the focus groups. Group A consisted of 5 people who had together completed the PT led intervention three months before the focus group, group B 6 people who had together just concluded their fitness instructor led programme, and group C 3 people who together had completed the PT led intervention four months before the focus group. The focus groups were held at the exercise venues which included hotels (A and B) and the MS Society building (C).

Ethical approval for this study was granted by the Scientific Research Ethics Committee in Limerick and the Clinical Ethics Committee of the Cork Teaching Hospitals.

## 4. Data Collection

Three focus groups were conducted by the same moderator with each focus group lasting approximately forty-five minutes and recorded using a digital recorder. An assistant moderator took notes throughout all three groups. Neither the moderator nor the assistant moderator had been involved in the RCT. The assistant moderator also observed group dynamics in order that nonverbal interaction taking place within the group could be linked to verbal accounts provided by the participants during analysis [20]. The questioning route used was of a conversational style with open-ended questions and is detailed as follows. Additional probes were used to supplement the questions.

*Question Route*

 Opening Question
  (i) To start with, we are just going to go around the room and introduce ourselves and where we are from.
 Introductory Question
  (i) So you have all spent 10 weeks participating in the “Getting the Balance Right” study; why did you decide to take part in it in the first place?
 Transitory Question
  (i) Have any of you had any previous experience of physiotherapy?
  (ii) Before starting the programme, what did you think you would get out of it?
 Key Questions
  (i) So now that you have completed the programme, how do you think it has affected you? Please tell me about both the positive and any negative effects.
  (ii) What aspect of the programme did you most enjoy?
  (iii) Do you see yourself continuing to exercise once the programme is completed?
 Summary
  (i) So, to sum up, if you had one thing to say about the programme, what would it be?
  (ii) Anything else you would like to add?

## 5. Data Analysis

At the conclusion of each group the assistant moderator delivered a summary of the focus group in order for the participants to confirm their interpretation of the discussion. The moderator and the assistant moderator then spent approximately thirty minutes in a debriefing session to capture their first impressions and highlight and contrast findings from previous focus groups [23].

Data were transcribed verbatim and analysed using thematic analysis using the methods described by Kreuger and Casey [24]. Transcripts were read and reread by both authors to become familiar with the data. Codes were developed independently and discussed and agreement was reached. Following this, themes were developed by the moderator (RC) and discussed and refined and agreement was reached with the assistant moderator (SC). To evaluate the weight of each theme, all associated quotes were grouped together and analysed for frequency (number of times a concept was discussed), extensiveness (number of participants who mentioned a concept), and intensity (the passion/emotion with which they discussed a concept) [24]. Following this procedure, the moderator created a list of “Big Ideas” and associated quotes. The assistant moderator read the transcripts to confirm the moderator's interpretation and verify the “Big Ideas.” Summaries of the transcripts were sent to all participants in order for them to confirm the moderator's interpretation of the focus groups. Nine of the participants confirmed the interpretation as accurate with no response from five. All of the above were done in order to confirm the credibility of the results and avoid selective perception.

## 6. Results

**Figure 1:**
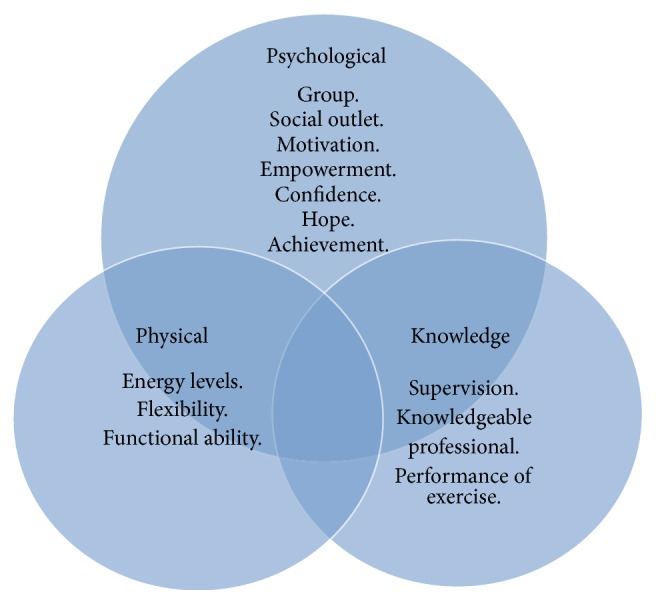
Themes.

Three focus groups were held with a combined number of 14 participants. Their mean age was 53.9 (SD13.0) and mean time since diagnosis was 10.3 (10.9) years. Eleven had relapsing remitting MS and 3 had secondary progressive MS. The participants in each focus group knew each other as they had been part of the same exercise groups. The group dynamic was lively and interactive across all three focus groups. All three groups were very passionate and positive about what they were talking about and this came across in their animated tone of voice and body language. The relative strength of these themes and their associated concepts are represented by the size of the circles in Figure 1. They are expanded below and accompanied by quotations that reflect the discussions and the context in which the themes arose. The quotes are preceded by the participants' group identification number.

The discussions in the focus groups were all extremely positive, with only one group raising a small negative view about the suitability of the hotel facilities for the exercise class. All other discussions focused on positive elements of the exercise classes.

Three common themes emerged from the analysis. These were the psychological benefits, the physical benefits, and the knowledge gained.

### 6.1. Psychological Benefits

One key element of the psychological benefits was the group and how the social aspects of group membership contributed strongly to their positive perceptions of the exercise class. They also talked about the “tips and advice” and the support that they gained from each other through exercising with people in similar situations: A4 “*for me the social thing was tremendous, meeting people, and meeting people who were in the same position as you*.” Participants talked frequently, extensively, and with emotion about how the group structure of the class served as a source of motivation and support. They talked about how it was much easier to exercise as a group rather than at home as the group motivated and supported each other. They also described the importance of the “team” and talked about how being a member of the team also contributed to motivating them to attend and to exercise at home. They talked about not wanting to let the team down and not wanting to get left behind: C1 “*but it's a great motivator to be in a group and if you're going into the group in the second week you're inclined to do the exercises so that you won't look, so that everyone else won't have gone way ahead of you*.”

The role of the group was reinforced by groups A and C who had finished the programme several months earlier. They talked about how the group aspect was very important to them as they now found it hard to motivate themselves to continue exercising. They were finding that without the support and discipline of the once a week classes they were finding it much more difficult to keep exercising: A2 “*I kind of miss that to be honest, that motivation*” and C3 “*You really have to dig deep to motivate yourself to do them [the exercises]*.”

In addition to the group serving to motivate and support them, participants found additional psychological benefits. These included empowerment and confidence and a sense of achievement and pride. A2 said “*I'd love to do it all over again, I've found that part of it, I'd love to do it all over again, you know as I say meeting everybody and every emotion and the sense of pride, I found all that good*.” The concept of empowerment arose frequently in all three groups and they talked about how it was important to them that this was something they could do for themselves. They felt that the programme encouraged them to take an active role in managing their illness and enabled them to put the effort into doing something for themselves. They also talked about how they felt empowered through the knowledge of what they could do for themselves and empowered by the achievement of completing the programme. B2 said “*It wasn't about taking a drug or a pill it was about helping yourselves*”; B1 “*Whereas this is exactly the opposite, this is people who have a problem who have said*” “*stuff my problem, I am going to march up the hill and not slide down it!*”

Participants spoke about how the programmes gave them a certain amount of control over their condition and an opportunity to try something in a “safe” environment. A2 said “*I'd be kind of worried about doing more damage than good, when I wouldn't know how to use the equipment or something like that*.” They also talked about how the programme had given them confidence to exercise themselves at the gym or at home and about how they would now consider going to places or doing activities they previously liked, which they would not have felt able to before. A4 said “*well I got a lot more confident, I was going completely introverted kind of*.”

### 6.2. Physical Benefits

The physical benefits that were most frequently talked about were the feelings of improved fatigue and increased energy and all participants were in agreement about these positive effects. These improvements in fatigue led in turn to increased ability to do things they could not do before. Several participants described “feeling lighter” to represent a feeling of reduced fatigue: A2 “*looking back I just be thinking about the amount of energy that it kind of gave me*”; B3 “*my energy … I'm not as tired, not as heavy, I used to feel so heavy in myself and dead in myself, I feel I'm lighter and more flexible*.”

Participants also reported improvements in hand or leg strength and flexibility which also led to an increased ability. B3 said “*well its good for our well being and we feel better that we're able to do things, because when we came in here we thought we wouldn't be able for any of this and now we're able for all this and keep up with the rest of the class*.”

The positive physical effects were described most frequently by group B who had finished their programme that day. They talked about seeing immediate improvements in energy and talked about how they felt better immediately after the exercise classes. Several people talked about how after the programme they had returned to valued hobbies such as gardening or had more energy to interact with grandchildren and family: C3 “*I found that this year was the first year in four years that I started doing a bit of gardening, it was a great feat for me to be able to finish it*.”

### 6.3. Knowledge

The role of knowledge in the positive perceptions of the programme was an emotive and extensive theme in all three focus groups. The majority of participants spoke about their limited knowledge before the programme of exercise for PwMS; they were afraid exercise might make their condition worse. Participants described a shift from thinking that exercise might do harm to knowing it was beneficial: A2 said “*I'd be kind of worried about doing more damage than good, when I wouldn't know how to use the equipment or something like that*” and C1 said “*the fact was that I loved walking and the fact that they thought I could walk, go for a walk again was a huge thing for me*.”

This knowledge came from two sources, the instructor and the actual completion of the exercises. They talked about the importance of a knowledgeable instructor who could advise how much or what to do when and about the need for the instructor to give feedback to ensure they were doing the exercises properly: C1 “*I mean you really need the professional guidance along the way to give you confidence and keep doing what you're doing and remind you of the benefit of it.*”

They also talked about now knowing what to do and what they could do, that is, the knowledge of their personal limits as well as what exercises were appropriate. The knowledge they now had also contributed to their confidence in their ability to continue exercising. B3 said “*we're motivated, we know the exercises to do now and we'll continue with those at home, because we know we're doing the right thing because she showed us the exercise.*”

## 7. Discussion

This study presents the qualitative analysis of the perceptions of participants from a multicentre, randomised, controlled trial of group exercise in the community. The role of the group as a positive aspect of the intervention crossed all themes. Three main themes emerged: the psychological benefits, the physical benefits, and the knowledge gained. While the physical benefits mirror the findings of other qualitative and quantitative exercise intervention studies, the additional information of the psychological benefits and knowledge gained can be used to inform the development and implementation of this and future interventions.

The role of the group as a positive aspect of the interventions is similar to qualitative findings following a strength training intervention. Dodd et al. [14] also found that the group interaction was a key external factor in programme completion. This has implications for the design and delivery of exercise interventions as the group has a key role in the positive perceptions of participants. The positive role of the group is mirrored by the qualitative evaluations of a group energy management [25] and cognitive [26] and fatigue management [27] interventions for PwMS. The “active components” or behaviour change techniques (BCTs) unique to group interventions can further highlight this positive role. Group interventions offer the possibility of BCTs such as facilitating social comparison and planning social support or change [28]. These BCTs have been proven successful in changing physical activity behaviour albeit in a healthy population [29]. As we further develop exercise interventions that aim to change PA behaviour in PwMS, it will be important to define and evaluate the contributions of these BCTs to exercise participation.

The role of the group as a motivating tool to enhance adherence and continued exercise after the programme is an important finding. Groups A and C were extremely vocal about how the group acted as a motivating tool for adhering to the programme during the ten weeks and stressed the difficulty they had in adhering to the exercises when the programme finished. McCullagh et al. [30] found that at a six-month follow-up to their exercise programme exercise capacity was not maintained due to the group not sustaining their level of exercise after the class and this is supported by the results of the trial [31]. Given the role of the peer group in motivating participants to exercise it may be necessary to explore ways for the exercise group to continue to meet with a lay facilitator, or peer facilitator, or indeed to use internet based [32] or teleconference based [33] methods in order to optimise continued adherence to exercise programmes without the continued need for direct professional supervision. Alternatively, further development and evaluation of the theory behind long term adherence to exercise for people with MS may be needed in order to develop interventions to promote long term exercise behaviour by people with MS that do not create “dependence” on the social support of the group but promote other behaviour change techniques such as goal setting and planning, feedback and monitoring, and self-belief. The effect of embedding the exercise intervention into a theory based behaviour change intervention is the focus of our group's next study [34].

Delivering interventions in groups as opposed to one on one may have significant financial implications; however, this has yet to be evaluated. It should be acknowledged that in all of these interventions participants volunteered to be part of a group intervention and this finding may therefore be unique for those with a preference for group interaction which may not be the case for all people with MS.

All participants reported significant psychological benefits from participating in the group exercise programme. There is limited information from previous exercise intervention studies to support the psychological benefits of exercise but this finding is mirrored by a statistically significant improvement on the MSIS-29v2 psychological scores in the main trial [35]. The exercise intervention was integrated with the group socialization; therefore it is difficult to separate the effects of enhanced fitness/strength from those of social interaction. Authors have described the “black box” that is physiotherapy intervention that combines both the* science* of exercise prescription and the* art* of programme delivery and social interaction and the difficulties associated with separating these factors. Future research should aim to control for contact and socialization in order to explore which aspect is driving the changes in psychological benefits.

Fatigue is common in MS with up to 80% of patients reporting it [36]. Recent systematic reviews suggest the positive effect of exercise on fatigue [12, 37] and this is matched by both these qualitative findings and from the quantitative evaluation of our trial [35]. Like those participants interviewed after a resistance training trial [14] our participants spontaneously brought up the positive effect on energy levels when asked about how the programme had affected them and talked about them frequently and with emotion. Fatigue and reduced mobility are strongly associated with unemployment and impact significantly on people's lives; interventions to reduce fatigue are therefore important. A recent meta-analysis [12] suggests that the pooled effect of pharmacological interventions for fatigue is only 0.07, while the pooled effect for exercise interventions is 0.57. This result combined with our qualitative and quantitative [35] data suggests that group, community based exercise is a promising intervention that may positively reduce the impact of fatigue.

Although there are many studies examining the effects that physical activity programmes can have on patients with MS, there is limited evidence examining the functional carryover of these programmes. Many studies report an increase in cardiovascular fitness and strength following exercise programmes [38, 39] but few investigate how these improvements affect the individuals in their day-to-day life. All three groups reported an increase in their functional capacity talking about the ability to participate in leisure hobbies such as gardening and walking and this then had a knock-on effect on their feelings of well-being. This highlights the positive addition of a qualitative analysis which found effects not captured by standardised outcome measures at participation level. The addition of participation measures to rehabilitation interventions in MS [40] warrants consideration in future studies.

The increase in knowledge experienced while participating in the group was a universal theme. The majority of participants spoke about their limited knowledge of exercise and its relationship with MS. This is something which they identified as being a barrier to their participation in exercise; they simply were afraid they may make their condition worse, a finding similar to that of Stuifbergen and Roberts [41]. Both these studies and the results of our qualitative analysis highlight the need for an education component during exercise sessions or a self-management approach to managing MS symptoms with exercise. Education and advice are important BCTs that have been shown to bring about improvements in PA behaviour in primary care interventions [42] and shaping knowledge and covert learning [29] are important behaviour change techniques that should be delivered in conjunction with attendance at an exercise class in order to facilitate long term physical activity behaviour change.

Linked to the need for education, participants reported that they felt the need for supervision, encouragement, and expertise of the instructor to enable them to achieve their programme goals. This is confirmed by the findings of Smith et al. [43] and Learmonth et al. [15] whose participants valued the support of a physiotherapist. Similarly, Wiles [44] suggests that verbal encouragement in the appropriate clinical context can have a major impact on perceived QOL and health status in patients with MS. They suggested that when delivering therapy the instructors can suggest pragmatic solutions to problems and provide general support to individuals who can be socially isolated. This suggests that supervised exercise programmes, where the instructor can both educate and provide verbal encouragement, may be initially required to optimise outcomes.

Some limitations to this study must be acknowledged. Although positive, these results are only applicable to those with a minor disability who use at most one stick to walk outdoors and only those who completed the trial were included. The question route sought to ask open questions to gain information on the positive and negative aspects of the programme; however, specifically asking about the parts they enjoyed may have introduced a positive bias.

The authors also acknowledge that it would have been beneficial to have carried out a second focus group with group B at their three-month follow-up and compare any changes in opinion from 1 day after conclusion to 3 months after conclusion. Additional interviews one year after intervention would also provide information on the long term adherence to the programme and should be considered in future studies.

Further exploration of the differences between physiotherapy and fitness instructor led groups is also warranted. While most themes were replicated across all groups, it is possible that data saturation did not occur and additional focus groups may have brought additional information.

Member checking was used to enhance the credibility of findings; however, lack of response from 5 participants may reduce credibility of the data.

## 8. Conclusion

The findings of the qualitative study suggest that participants in a group exercise programme perceive significant benefits. The group environment was deemed a vital component of the programme and had important implications for adherence and motivation especially once the programme reached its conclusion. The qualitative evaluation reinforces the findings from the quantitative study and brings to the table new information on the positive effect of group exercise on fatigue. This qualitative data also demonstrates how physical improvements translate to domestic life for people with minimal disability due to MS. This study highlights some of the exercise issues that are pertinent to MS patients and should help inform clinicians when designing a group exercise programme that has transferability to the home environment.

## Implications for Rehabilitation

- Qualitative analysis of community based exercise suggests that the role of the group may be a key factor in the psychological benefits and may aid motivation.
- Physical improvements that translate to domestic life and reductions in fatigue were reported.
- Education and supervision are important; participants reported increased knowledge which shifted from a fear that exercise might do harm to an appreciation of the benefits of exercise.
